# EDITORIAL COMMENT: OFF-CLAMP ROBOTIC-ASSISTED PARTIAL NEPHRECTOMY

**DOI:** 10.1590/S1677-5538.IBJU.2015.0386.1

**Published:** 2016

**Authors:** Mike Bozin, Homayoun Zargar

**Affiliations:** 1 Peter MacCallum Cancer Centre, Melbourne, VIC, Australia; 2 Royal Melbourne Hospital, Melbourne, VIC, Australia; 3 Australian Prostate Cancer Research Centre, Melbourne, VIC, Australia

In this video the Wright et al. (1) demonstrate their off-clamp technique of robotic-assisted partial nephrectomy (RAPN) and subsequently report the outcomes of a cohort of patients previously treated utilizing this technique. The method of choice for haemostasis in this video is electrocautery with the compression of larger vessels with robotic fourth arms if troublesome bleeding is encountered. One assumes if further bleeding is encountered, haemostasis can also be obtained using suture ligation. Off-clamp RAPN has gained popularity in recent years with the view to eliminate warm ischemia in order to maximize preservation of renal function. Various techniques for achieving this goal have been described ranging from tertiary and quaternary branch dissection to utilization of preplaced sutures in selected cases at the time of RAPN (2, 3). Zero ischemia comes at the potential price of increase in blood loss, higher complication rates and inferior visualization of tumor bed compromising resection with negative surgical margin. However with technical refinements and an increase in cumulative experience such drawbacks can be eliminated as demonstrated in this series (4).

On the flipside evidence from studying changes in the operated kidney using MAG3 renal scan also supports the notion that not every minute of ischemia counts and renal parenchyma preservation plays a more important role in renal function preservation if the warm ischemia time can be limited to less than 25-30 minutes (5, 6). Furthermore where prolonged ischemia time anticipated intracorporeal cooling could mitigate the deleterious effects of warm ischemia without compromising renal functional outcomes (7). Future studies need to explore the role of renal ischemia preconditioning, use of novel renoprotective agents, as well as quantifying the influence of unmeasured factors on renal function such as the impact of local ischemia caused by renorrhaphy.

*Homayoun Zargar, MD* *Department of Urology* *Royal Melbourne Hospital* *300 Grattan St, Parkville* *VIC 3050, Australia* *E-mail: homi.zargar@gmail.com*
